# Local and Systemic Antibiotics in Peri-Implantitis Management: An Umbrella Review

**DOI:** 10.3390/antibiotics12010114

**Published:** 2023-01-08

**Authors:** Giovanni Boccia, Federica Di Spirito, Francesco D’Ambrosio, Maria Pia Di Palo, Francesco Giordano, Massimo Amato

**Affiliations:** Department of Medicine, Surgery and Dentistry, University of Salerno, 84081 Baronissi, Italy

**Keywords:** peri-implant disease, peri-implantitis, antibiotic, antibiotics, local, systemic, locally-delivered, systemically-delivered

## Abstract

The present umbrella review aimed to characterize the type and regimen of antibiotics administered locally and/or systemically, alone or in combination with surgical and nonsurgical treatments, for peri-implantitis and to evaluate and compare the associated clinical, radiographic, and crevicular peri-implant outcomes. The secondary objective was to determine the most effective antibiotic type, route of administration, regimen, and protocols (antibiotics alone or in combination with other approaches) for treating peri-implantitis. The study protocol, which was developed in advance under the PRISMA statement, was registered at PROSPERO (CRD42022373957). BioMed Central, Scopus, MEDLINE/PubMed, the Cochrane Library databases, and the PROSPERO registry were searched for systematic reviews through 15 November 2022. Of the 708 records found, seven reviews were included; three were judged of a critically low and four of low quality through the AMSTAR 2 tool. Locally administered antibiotics alone or as an adjunct to surgical or nonsurgical treatments for peri-implantitis showed favorable outcomes, albeit with limited evidence. The administration of systemically-delivered antibiotics in combination with nonsurgical or surgical treatments remained questionable. Local plus systemic antibiotics have not been shown to have durable efficacy. Due to the heterogeneity of reported antibiotic types, routes, regimens, and protocols, no definitive conclusions could be drawn regarding the most effective antibiotic use in treating peri-implantitis.

## 1. Introduction

Dental implants have become one of the most reliable therapeutic options to replace missing teeth. As a result, a significant increase in the number of dental implants placed annually, on the one hand, and in the number of cases diagnosed with peri-implantitis, on the other hand, has been observed worldwide [1,2].

The 2017 World Workshop on the Classification of Periodontal and Peri-Implant Diseases and Conditions defined peri-implantitis as a pathological condition occurring in the tissues surrounding dental implants characterized by signs of inflammation of the outer tissues and progressive bone loss [3,4,5,6,7,8].

A number of therapeutic approaches have been proposed for peri-implantitis management [9,10,11,12,13,14,15,16,17,18,19], including nonsurgical, surgical, and combined treatments. Most of the peri-implantitis treatments are also performed for periodontitis because of the common etiology [3,4,5,6,7,8], and the similarities in the pathophysiology of bacterial biofilm formation on both dental implant surfaces and dental tissues [12].

Abrasive air powder, metallic or non-metallic curettes (the latter made of carbon or plastic, reinforced or not with resin), with an ultrasonic scaler with a metal or plastic tip [14,15] provide nonsurgical mechanical biofilm control. In addition, implantoplasty is performed to reduce the roughness of dental implant surfaces and, in turn, bacterial adhesion [15]. Further physical methods, such as phototherapy and laser therapy, e.g., with a continuous carbon dioxide laser, have also been proposed in peri-implantitis management [16,17].

As a counterpart, chemical biofilm control mainly relies on chlorhexidine, along with hydrogen peroxide, cotton pellets soaked in saline solution and citric acid [12].

However, nonsurgical treatment has not yet shown fully predictable results in peri-implantitis treatment [9,10], and long-term data on outcomes after surgical treatment show only a slight improvement in bone levels [10,11].

Moreover, considering that the microbial flora characterizing peri-implantitis has a broader spectrum compared to periodontitis and that the biofilm of peri-implantitis is characterized by higher counts of human cytomegalovirus and Epstein–Barr virus [7,8,13], reducing the number of pathogens and changing the bacterial biofilm composition might be even more critical in peri-implantitis management. From this point of view, the control of biofilm and the reversal of dysbiosis could benefit from the administration of probiotics [8].

Furthermore, antibiotics administration as adjuncts to peri-implantitis treatment was first proposed by Mombelli and Lang in 1992 [18]. Since then, several studies have been conducted to evaluate the beneficial effects of systemically- and locally-delivered antibiotics in combination with other treatments [19]. However, it is still controversial whether the concomitant systemic or local use of antibiotics is beneficial in treating peri-implantitis [19]. In addition, since antibiotics are among the most commonly prescribed medications by dentists, often without precise indications, thus violating antibiotic stewardship [20,21,22,23], their use should be appropriately evaluated.

Therefore, the present umbrella review aimed to characterize the type and regimen of antibiotics administered locally and/or systemically, alone or in combination with surgical and nonsurgical treatments for peri-implantitis, and to evaluate and compare the associated clinical, radiographic, and crevicular peri-implant outcomes. The secondary objective was to determine the most effective antibiotic type, route of administration, regimen, and protocols (antibiotics alone or in combination with other approaches) for treating peri-implantitis.

## 2. Materials and Methods

### 2.1. Study Protocol

Prior to the literature search, data extraction, and analysis, the study protocol was defined under the Preferred Reporting Items for Systematic Reviews and Meta-analyses (PRISMA) statement [24], and registration was made in the PROSPERO Registry of Systematic Reviews (CRD42022373957) [25].

The questions were formulated based on the PICO model [26,27], and record search and study selection strategies were developed.

The research question [28] focused on the efficacy of type, routes of administration, and regimens of locally- and/or systemically-delivered antibiotics alone or in combination with other (surgical or nonsurgical) peri-implantitis treatments for improving peri-implant outcomes, specifically:

P-Population: subjects with at least one dental implant and implant-supported restoration(s) with peri-implantitis:

I-Intervention: locally and/or systemically administered antibiotics alone or (all);
C-Comparison: no intervention, placebo, between different interventions (different type, routes of administration, and regimens of locally- and/or systemically-delivered antibiotics alone or in combination with other surgical or nonsurgical peri-implantitis treatments);
O-Outcome(s): clinical and radiographic and crevicular peri-implant parameters.

### 2.2. Search Strategy

Systematic reviews (with or without meta-analysis) published in English on local and/or systemic antibiotics in peri-implantitis treatment were searched electronically through the PROSPERO registry and Scopus, MEDLINE/PubMed, BioMed Central, and Cochrane Library databases by three independent reviewers (F.D.S., F.D.A., M.P.D.P.) without date restriction until 15 November 2022, using keywords and Boolean operators as follows: (“peri-implantitis” OR “peri-implant dis-ease” OR “dental implant” OR “implant loss”) AND (“local antibiotic” OR “local antibiotics” OR “general antibiotic” OR “general antibiotics” OR “antibiotic therapy” OR antibiotics OR antimicrobial OR “peri-implant mucositis”).

The following filters were applied: “review (English)” and “refine”: “review (English)” in the Scopus database; “systematic review (English)” in the MEDLINE/PubMed database; “keywords” in the Cochrane Library; no filters were used in the BioMed Central database or the PROSPERO registry.

### 2.3. Study Selection and Eligibility Criteria

Collected citations were recorded, duplicates were eliminated via the reference management tool EndnoteTM (Clarivate), and the remaining systematic review titles (with or without meta-analysis) were screened by the three independent reviewers (FDS, FDA, MPDP), who then screened relevant abstracts.

The full texts of these potentially eligible title-abstracts were obtained, contacting the study authors if full texts were unavailable, and were independently reviewed by the same authors (FDS, FDA, MPDP). Any differences of opinion were clarified by consultation, and in case of doubt, another author (FG) was consulted. No manual search was carried out from the reference lists of included articles.

Inclusion criteria: no restrictions were applied on publication date, the number of studies, and study design included in each systematic review; age, gender, and characteristics of the participants; the number of dental implants and type of prosthetic restorations; type and regimen of antibiotics administered locally and/or systemically, alone or in combination with other (nonsurgical or surgical) peri-implantitis treatment.

Exclusion criteria: non-English language studies and self-reported peri-implant status were excluded.

### 2.4. Data Extraction and Collection

Data were extracted independently and in duplicate by two authors (F.D.A., M.P.D.P.) using a standardized data extraction form developed based on the models recommended for intervention reviews of RCTs and non-RCTs [29] before data extraction; a third author (F.D.S.) was consulted in case of disagreement.

Of each systematic review (with or without meta-analysis) included in the present umbrella review, data illustrated in Table 1 were recorded.

### 2.5. Data Synthesis

A narrative synthesis of the data regarding the population studied, intervention(s), and outcomes was performed.

Data from the included studies were qualitatively synthesized:

- to characterize the type and regimen of antibiotics administered locally and/or systemically alone or in combination with other (surgical or nonsurgical) peri-implantitis treatments and comparisons;
- to assess clinical, radiographic, and crevicular peri-implant outcomes according to the type and regimen of locally- and/or systemically-delivered antibiotics administered alone or in combination with other (surgical or nonsurgical) peri-implantitis provided;
- to compare clinical, radiographic, and crevicular peri-implant outcomes after administration of locally- and/or systemically-administered antibiotics alone or in combination with other (surgical or nonsurgical) peri-implantitis vs. placebo and to each other.

### 2.6. Quality Assessment

The quality assessment of the systematic reviews included in this umbrella review was performed using the Assessing the Methodological Quality of Systematic Reviews (AMSTAR) 2 tool, accessed online (https://amstar.ca) on 16 November 2022, evaluating for quality the systematic reviews with or without meta-analysis [30].

## 3. Results

### 3.1. Study Selection

A total of 708 records were identified from the electronic search, specifically 64 from BioMed Central, 216 from Scopus, 252 from MEDLINE/PubMed, five from the Cochrane library databases, and 171 from the PROSPERO register.

In total, 330 duplicates were eliminated, and 378 title-abstracts were screened.

Of these 378 title-abstracts, 17 abstracts were relevant and compliant with the eligibility criteria of the present systematic review.

Full texts were screened, and 10 articles were further excluded, specifically because: (n = 4) not relevant; (n = 1) describing antibiotic use combined with antiseptic agents in implant surgery; (n = 1) antibiotics were not used in peri-implantitis treatment; (n = 2) describing prophylactic antibiotic use; (n = 2) reviews were still ongoing (Table 2).

**Figure 1:**
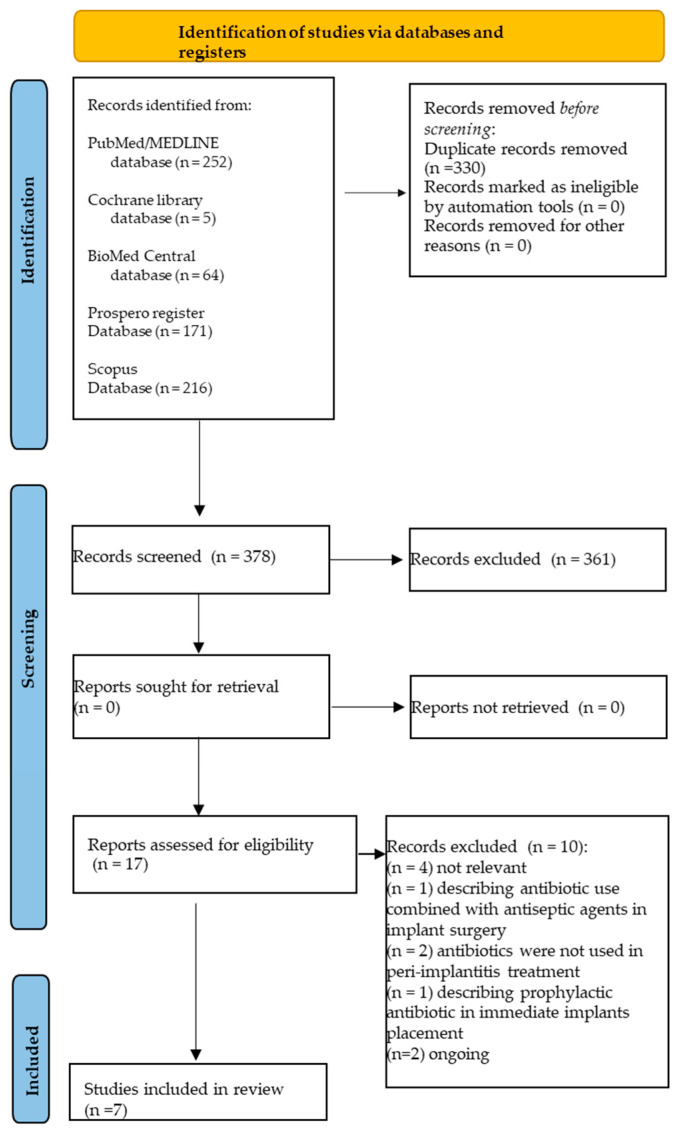
Study selection flowchart.

A total of seven systematic reviews [41,42,43,44,45,46,47], four with meta-analysis [41,43,45,47], were finally included in the present umbrella review (Figure 1). The seven systematic reviews [41,42,43,44,45,46,47] included 42 randomized controlled trials (RCTs) [41,42,43,44,45,46,47], 10 prospective studies (PS) [41,45], seven systematic reviews (SR) [44], four case series (CS) [41,43], three case-control studies (CCS) [41], and two studies whose typology was not defined [47].

### 3.2. Study Characteristics

The total number of subjects involved was 1575 with 2262 implants. However, three studies [44,46,47] did not report the sample size or the number of implants. In no case was the gender ratio or mean age of participants or the number, location, characteristics, and survival of implants affected by peri-implantitis reported.

The characteristics and outcomes from included studies are synthesized in Table 3.

### 3.3. Local and Systemic Antibiotics in Peri-Implantitis Management

Locally-delivered antibiotics for treating peri-implantitis were administered alone and in combination with other nonsurgical and surgical interventions and systemic ones.

No data were available about the characteristics of systemic antibiotic therapy alone in treating peri-implantitis from included studies. 

#### 3.3.1. Locally-Delivered Antibiotics Alone and in Combination with Nonsurgical and Surgical Treatment of Peri-Implantitis

Three systematic reviews [41,42,46] described findings from studies with local antibiotic administration combined or not to other peri-implant treatments.

Local minocycline (“Arestin” in microspheres, “Periocycline” in ointment), doxycycline gel (“Atridox”, “Ligosan”), lincomycin gel, wrythromycin gel, retracycline fibers “Actisite”, and metronidazole gel “Elyzol” proved to reduce PPD and BoP [41]. However, local minocycline microspheres [42] and metronidazole gel 25% [46] did not positively affect either BoP, PPD, and PI [42], or implant failure [46], respectively.

Four systematic reviews [42,43,46,47] synthesized peri-implant outcomes following locally-delivered antibiotics combined with other interventions.

Doxycycline hyclate 8.5% + SRP, minocycline 10 mg in 0.5 g of ointment + surgical treatment, and minocycline ointment + nonsurgical treatment improved BoP, GI, PPD, and PI at 4–6 months follow-up [42].

Local tetracycline hydrochloride delivery by monolithic ethylene vinyl acetate fiber (for one time of antibiotic with a duration of 10 days) + rubber cup polishing + SRP, doxycycline “Atridox” subgingivally for one time + SRP and irrigation with 0.2% CHX, and minocycline “Periocline” subgingivally + OFD at 1, 3, and 6 months were found to positively affect PPD and BoP up to 12 months after therapy [43].

Local doxycycline hyclate 8.5% “Atridox” applied through a syringe with a blunt cannula in the peri-implant sulcus + SRP determined a more significant improvement in CAL and PPD values compared to mechanical debridement at 4 months follow-up [46].

Local minocycline gel + ultrasonic periodontal debridement, minocycline hydrochloride microspheres + SRP, and metronidazole (400 mg) + amoxicillin (500 mg) + SRP equally decreased PPD, BoP, and CAL values compared to the baseline [47].

Characteristics of antibiotic administration in peri-implantitis treatment are detailed in Table 4 for locally-delivered antibiotics administered alone (not in combination with nonsurgical and surgical treatments) and in Table 5 for locally-delivered antibiotics combined with other interventions.

#### 3.3.2. Systemically-Delivered Antibiotics in Combination with Nonsurgical and Surgical Treatment of Peri-Implantitis

Four systematic reviews [43,44,45,47] reported results from systemically-delivered antibiotics, which were always used in combination with other interventions in treating peri-implantitis.

Several drug regimens were recorded for amoxicillin (750 mg/12 h or 500 mg/8 h for 7 d, or 500 mg/24 h at 1 std and 250 mg/24 h for 2–4 d + mechanical implant surface debridement; 750 mg/12 h for 3 d preoperatively and 7 d postoperatively + open flap debridement + resective techniques), azithromycin (250 mg/12 h on the d of surgery + 250 mg/24 h for 4 d + open flap debridement, or 500 mg/24 h for 3 d + full mouth scaling and root planing), metronidazole (250 mg/8 h for 7 d + nonsurgical debridement 500 mg/8 h for 7 d + mechanical implant surface debridement), amoxicillin (500 mg/8 h for 7 d) + metronidazole (400 mg/8 h for 7 d) for 5 to 7 days in combination with nonsurgical treatment, open flap debridement and mechanical implant surface debridement, as well as for other antibiotic combinations (e.g., clindamycin + metronidazole + azithromycin + tetracicline for 4 weeks and metronidazole + amoxicillin + ciprofloxacin + sulfonamide + trimethroprim + metronidazole for 2 weeks), overall highlighting that systemic antibiotics should be carefully evaluated in peri-implantitis management considering the risk of antibiotic resistance [45].

Azithromycin (500 mg on 1 d and 250 mg on 2 and 4 d +/− scaling and root planing + rubber cup polishing +/− open flap debridement; 500 mg/d for 3 d preoperatively + scaling and root planing), amoxicillin (1.5 g for 3 d preoperatively and 7 d postoperatively + open flap debridement + bone recontouring + rubber cup polishing + chlorhexidine 0.2%), and amoxicillin (500 mg/8 h for 14 d) + metronidazole (400 mg/24 h for 14 d) provided benefits in clinical peri-implant outcomes for up 12 months after therapy [43]. In contrast, amoxicillin (750 mg/12 h + chlorhexidine 0.2%+ mechanical implant surface debridement) and azithromycin (250 mg/12 h for 2 d and 250/24 h for 4 d) did not show beneficial effects [44].

The association of amoxicillin and metronidazole in combination with ultrasonic debridement (500 mg/8 h + 500 mg/24 h for 7 d), scaling and root planing (375 mg/8 h for 7 d + 250 mg/8 h for 7 d; 500 mg/8 h + 400 mg/24 h for 7 d), as well as the administration of clarithromycin in combination with the antimicrobial photodynamic therapy (500 mg/24 h for 3 d) significantly reduced BoP, CAL, and PPD [47].

Table 6 synthesizes the characteristics of systemically-delivered antibiotics combined with other interventions in the treatment of peri-implantitis.

#### 3.3.3. Locally- Plus Systemically-Delivered Antibiotics in Combination with Nonsurgical and Surgical Treatment of Peri-Implantitis

Two systematic reviews [39,43] reported peri-implant outcomes after local plus systemic antibiotics administration alone or combined with other interventions in treating peri-implantitis.

Local minocycline “Periocline” + amoxicillin (500 mg/8 h for 3 d) in combination with open flap debridement + scaling and root planing at 1, 3, 6 months proved some benefits in BoP and PPD values [43].

Local metronidazole 25% gel “Elyzol” associated with tetracycline hydrochloride “Ambramicine” in combination with apically repositioned flap, as well as amoxicillin (50 mg/kg/d for 8 d) in combination with SRP before surgery did not affect peri-implant outcomes at 2-year follow-up [39].

Table 7 shows the characteristics of local plus systemic antibiotics administered alone or in combination with other interventions in treating peri-implantitis.

### 3.4. Quality Assessment

Three of the studies were judged of critically low [42,46,47] and four of low quality [41,43,44,45] through the Assessing the Methodological Quality of Systematic Reviews (AMSTAR) 2 tool [30], as illustrated in Table 3.

## 4. Discussion

The treatment of peri-implantitis, which aims to reduce the microbial load, decontaminate the dental implant surface, and eliminate peri-implant mucosal inflammation, thereby preserving the peri-implant bone, can be divided into nonsurgical approaches using mechanical instrumentation, antibiotics, antiseptics, and chemical or laser decontamination of the dental implant surface and surgical approaches such as air powder abrasion, resective, or regenerative procedures [48,49,50,51,52].

Nonsurgical treatment of peri-implantitis aims to reduce inflammation by controlling biofilm [50]. Mechanical therapy includes using resin, carbon, or titanium cuvettes, specialized ultrasonic instruments, rubber bowls/polishing brushes, and air-powder devices [50]. However, nonsurgical mechanical debridement alone is ineffective for the long-term treatment of peri-implantitis [50]. Adjunctive antiseptic agents such as chlorhexidine improve clinical parameters but only for six months or less [50].

Surgical treatment of peri-implantitis is required when PPD and bone resorption have progressed or persist in removing biofilm and creating a surface that allows re-osteointegration through a full-thickness flap [50]. Accordingly, in conjunction with open-access flaps, mechanical debridement and adjuvants, such as saline, abrasive pumice, citric acid, chlorhexidine, and hydrogen peroxide, are used [50]. The open approach and the morphology of the residual bone determine the choice of surgical intervention and whether a resective or regenerative approach is opted for [53].

Given these considerations and the fact that peri-implantitis is maintained by microorganisms [48], it is legitimate to ask whether systemic and/or local antibiotics are effective in treating peri-implantitis [42]. Accordingly, concomitant antibiotics, including minocycline and doxycycline hyclate, have improved gingival inflammation and probing depths [50]. However, further studies are needed to verify their long-term efficacy.

Therefore, the present umbrella review aimed to characterize the type and regimen of antibiotics administered locally and/or systemically, alone or in combination with surgical and nonsurgical treatments for peri-implantitis, and to evaluate and compare the associated clinical, radiographic, and crevicular peri-implant outcomes. The secondary objective was to determine the most effective antibiotic type, route of administration, regimen, and protocols (antibiotics alone or in combination with other approaches) for treating peri-implantitis.

### 4.1. Locally-Delivered Antibiotics Alone and in Combination with Nonsurgical and Surgical Treatment of Peri-Implantitis

The efficacy of locally-delivered antibiotics alone on periodontal parameters, especially PPD, BoP, and plaque index (PI), was investigated in three studies [41,42,46] with contrasting results, probably due to the different interventions and control groups in each of the three studies [41,42,46].

Indeed, only one study [41] found an improvement in PPD and BoP after using local antibiotics compared with the control group. Toledano et al. [41] demonstrated that the local use of antibiotics in peri-implantitis patients positively affected the reduction of PPD and BoP. Specifically, the likelihood of bleeding on probing was halved when antibiotics were applied topically, and on average, a 0.30 mm reduction in PPD was observed when topically administered antibiotics were used [41]. However, peri-implant parameters in subjects under minocycline [41] were not significantly different from those using chlorhexidine [42]. Similarly, metronidazole was reported to be associated with both improvements in peri-implant conditions [41] and implant failure in the studies by Esposito et al. [46]. 

Four studies investigated the efficacy of local antibiotics in combination with nonsurgical and surgical treatment of peri-implantitis. Except for one study in which implant failure was recorded [46], the various combinations of tetracyclines (doxycycline or minocycline) with nonsurgical treatments (such as scaling and root planing and photodynamic therapy) or surgical flap incision always resulted in improvement of peri-implant BoP and PPD [42,43,47], even at a follow-up of 12 months [43]. Clinical attachment loss (CAL) [47] and gingival index (GI) and PI also improved in some cases [43].

Accordingly, Passarelli et al. [42] found an improvement in PPD and BoP when local antibiotics were combined with nonsurgical treatment compared with nonsurgical treatment alone [42].

Tetracyclines were the most commonly administered antibiotics [41,42], especially doxycycline and minocycline, which are also the most commonly used in treating periodontal infections (Rodrigues, 2004), followed by metronidazole, which showed a positive effect either if used alone in comparison with photodynamic therapy [42] or in combination with amoxicillin and scaling and root planing [47].

### 4.2. Systemically-Delivered Antibiotics in Combination with Nonsurgical and Surgical Treatment of Peri-Implantitis

Systemically-delivered antibiotics have always been evaluated in combination with other interventions, likely because antibiotic stewardship advises against prescribing antibiotics unless they are indispensable and their efficacy is supported by evidence to combat antimicrobial resistance [20,22].

A total of four studies evaluating the efficacy of systemic antibiotics in combination with nonsurgical and surgical treatment for peri-implantitis were presently retrieved.

Different associations of amoxicillin and metronidazole at variable doses and durations were administered in combination with nonsurgical (such as scaling and root planing, nonsurgical debridement) and surgical treatments with implant debridement and showed improvement in PPD and BoP in only two studies [43,47]. Conversely, Toledano-Osorio et al. [45] showed that systemically-delivered administered antibiotics did not significantly improve BoP and PPD, although improvements in CAL, suppuration, recession, and total bacterial count were described in patients with peri-implantitis [45]. Similarly, Oen et al. [44] found no improvement in PPD when systemic antibiotics were combined with surgical procedures.

Consequently, combining systemically-administered antibiotics with nonsurgical and surgical treatments for peri-implantitis remains controversial in the current state of knowledge. However, there is some evidence to support their use in recurrent and refractory peri-implantitis cases [35]. 

### 4.3. Efficacy of Locally-Plus Systemically-Delivered Antibiotics in Combination with Nonsurgical and Surgical Treatment of Peri-Implantitis

The efficacy of locally- plus systemically-delivered antibiotics in combination with nonsurgical and surgical treatment was investigated in two studies [39,43].

Wang et al. [43] concluded that using local plus systemic antibiotics in combination with other peri-implantitis treatments had a beneficial effect and significantly improved PPD values [43]. However, the authors suggested that such a positive result could also be attributable to the combination of local and systemic administration of antibiotics with open flap debridement and scaling and root planing [43].

Esposito et al. [39] also reported the concomitant use of systemic and local amoxicillin, with no significant improvement in PPD, CAL, and REC from baseline at 2-year follow-up. Therefore, despite the limited data, local plus systemic administration of amoxicillin may have no durable efficacy.

### 4.4. Local and Systemic Antibiotics in Peri-Implantitis Management: Clinical Considerations

Nonsurgical treatments such as mechanical debridement and oral hygiene instructions effectively managed peri-implant mucositis and reduced but did not eliminate the inflammatory signs and symptoms of peri-implantitis [42,54,55,56]. In the latter case, the surgical treatment allows direct decontamination of the dental implant surface and complete removal of the inflammatory granulation tissue [55].

Chemical biofilm control and locally- and systemically-delivered antibiotics administration have been proposed to improve nonsurgical and surgical treatment outcomes.

In particular, although supported by mild evidence, locally-delivered antibiotics used as an adjunct to nonsurgical mechanical debridement produced favorable results. In addition, local antibiotics, in combination with other surgical interventions, also showed modest clinical improvements in peri-implant parameters. Because adequate drug concentration at the site of infection is required for a sufficient time to eliminate the pathogens [57], various drug carriers have been developed to improve outcomes associated with locally-delivered antibiotics.

Conversely, systemically-delivered antibiotics had no significant effect on the treatment of peri-implantitis, either in combination with nonsurgical or surgical treatments [43,44,45,47]. As there is currently no clear indication for the use of systemically-delivered antibiotics for treating peri-implantitis, their administration should be evaluated even more cautiously, given the ever-growing phenomenon of antimicrobial resistance [45].

In fact, dentists are responsible for 10% of all antibiotic prescriptions [58], and antimicrobial resistance is mainly caused by the overuse and misuse of antibiotics [59], leading to an increased risk of complications and mortality and a more complex resolution of infectious diseases [20,22,60]. In this perspective, alternative techniques to avoid or reduce antimicrobial administration have been introduced as an adjunct to nonsurgical and surgical treatment of peri-implantitis, such as photodynamic therapy (aPDT). Specifically, Zhao et al. [47] reported promising results after aPDT comparable to those of antibiotic administration, although stating that further studies are needed to evaluate the efficacy of aPDT as an alternative to antibiotics [47].

Due to the variability of antibiotic types, routes of administration, dosages, and combinations with other nonsurgical and surgical interventions, the most effective antibiotic type, route of administration, regimen, and protocol (antibiotics alone or in combination with other approaches) for the management of peri-implantitis could not be determined from the currently retrieved data for either locally- or systemically-delivered antibiotics.

Moreover, given the conflicting results and following antibiotic stewardship to combat antimicrobial resistance [20,22,60,61,62], it may be concluded that the prescription of systemically-delivered antibiotics should be discouraged, and the use of locally-delivered ones should be cautiously evaluated based on a case- and site-specific benefit-risk assessment.

Further research on the type of antibiotic, route of administration, therapeutic regimen, and protocols (antibiotics alone or in combination with other approaches) for managing peri-implantitis is still needed to demonstrate the most effective antibiotic therapy and to limit the risks resulting from inappropriate antibiotic therapy.

Given the heterogeneous data on antibiotic type, routes of administrations, therapeutic regimens, duration, and combination with other interventions, the incomplete data on peri-implant outcomes, and those missing on dental implants’ number, location, characteristics, and survival, no meta-analysis could be conducted. Findings from the presently included systematic reviews revealed contrasting and inconclusive results concerning the effectiveness of systemically- and locally-delivered antibiotics in peri-implantitis management. As a result, the ideal antibiotic type, route of administration, regimen, and protocols (antibiotics alone or in combination with other approaches) in peri-implantitis management could not be determined.

In addition, some studies, such as those by Renvert et al. and Cha et al. [63,64], were included in multiple systematic reviews, and all included reviews were classified as of low [41,43,44,45] or critically low quality [42,46,47]. Consequently, the need for future investigations with higher evidence levels, such as randomized controlled trials, with defined antibiotic therapeutic regimens and protocols, methodically recorded peri-implant outcomes, and at least > 3 years of follow-up [44] are needed. Moreover, the need for case- and site-specific indication of antibiotic administration and benefit-risk assessment should be provided.

However, the present umbrella review may be the first to collectively evaluate the effectiveness and the proposed regimens and protocols of local and/or systemic antibiotic administration alone and in combination with other nonsurgical or surgical peri-implantitis treatments.

## 5. Conclusions

Seven systematic reviews were included in the present umbrella review; three were classified as critically low quality and four as low quality using the AMSTAR 2 tool.

Locally-delivered antibiotics administered alone or as an adjunct to surgical or nonsurgical treatments for peri-implantitis showed favorable results, albeit with limited evidence. The administration of systemically-delivered antibiotics in combination with nonsurgical or surgical treatments remained questionable. Local plus systemic antibiotics have not been shown to have durable efficacy.

Because of the heterogeneity of reported antibiotic types, routes, regimens, and administration protocols, no definitive conclusions could be drawn regarding the most effective antibiotic use in managing peri-implantitis. However, given the phenomenon of antimicrobial resistance, the prescription of systemically-delivered antibiotics should be discouraged, and locally-delivered ones should be used with caution based on a case- and site-specific benefit-risk assessment.

Further research is needed on antibiotics’ type, route of administration, therapeutic regimen, and protocols (antibiotics alone or in combination with other approaches) for managing peri-implantitis, pointing out the case- and site-specific indications and the most effective antibiotic therapy, concurrently limiting the risks of inappropriate antibiotic use.

## Figures and Tables

**Table 1:** Data extracted and collected from the systematic reviews included in the present umbrella review.

Data Extracted and Collected from the Systematic Reviews Included in the Present Umbrella Review	
Systematic reviews	Authors, Year
	Journal
	Meta-analysis
	Funding
	Quality
	Conclusions
Studies included in the systematic reviews	Characteristics
	(number and design)

	Population
	(sample size, mean age, gender ratio)

	Peri-implantitis sites
	(dental implants’ number, position, and survival)
Administered antibiotics	Local or systemic antibiotics
	(type, delivery vehicle, regimen, duration)
	Combined peri-implant treatment (if any)
	(type, sessions, follow-up)
Peri-implant outcomes (statistically significant)	Clinical parameters
	Radiographic parameters
	Crevicular parameters

**Table 2:** Studies excluded and reasons.

Authors, Year	Reason for Exclusion
Alenzi, A., 2020 [31]	Not pertinent
Rodríguez Sánchez, 2018 [32]	Not pertinent
Grusovin, M. G., 2010 [33]	Not pertinent
Caiazzo, 2021 [34]	Antibiotics used in combination with antiseptics in implant surgery
Khan, 2020 [35]	Antibiotics were not used in peri-implantitis treatment
Salgado-Peralvo, 2021 [36]	Antibiotics used as prophylaxis in immediate implants placement
Bizelli, 2020 [37]	Review ongoing
Soo Jim Lin, 2018 [38]	Review ongoing
Esposito, M., 2003 [39]	Not pertinent
Ata-Ali, 2013 [40]	Antibiotics were used in postoperative infections and implant failure

**Table 3:** Characteristics and outcomes from included studies. Source: first author, year, reference, journal of publication, meta-analysis, funding, and study quality (if any). Studies reported the systematic reviews included in the present umbrella review: design and number; population sample size (n.), mean age (y.o.), and gender ratio (M/F); dental implant number, position, and survival. Type, route of administration, regimen, and duration of locally- and/or systemically-delivered antibiotics alone or in combination with other (surgical or nonsurgical) peri-implantitis treatment. Clinical, radiographic, and crevicular peri-implant outcomes (statistically significant). Conclusions.

Authors, Year Reference Journal Meta-Analysis Funding Quality	Studies (Number and Design) Population Sample Size Mean Age Gender Ratio Peri-Implantitis Sites (Dental Implants’ Number, Position, and Survival)	Administered Antibiotics Local or Systemic (Type, Delivery Vehicle, Regimen, Duration) Combined Peri-Implant Treatment (Type, Sessions, Follow-Up)	Peri-Implant Outcomes (Statistically Significant) Clinical Parameters Radiographic Parameters Crevicular Parameters	Conclusions
Toledano, 2021 [41] Journal of Dent Systematic review and Meta-analysis Low quality	Studies: n.12 RCT (n.7) CS (n.1) CCS (n.3) PS (n.1) Population sample size n.365 Implants n.463 Mean age: MD Gender ratio: MD Dental implants affected Number: MD Position: MD Characteristics: MD Survival: MD	Local antibiotics: yes Type: minocycline, doxycycline, lincomycin, erythromycin, tetracycline Delivery vehicle: gel, microspheres, fibers, powdered, bone graft, ointment Duration: 4, 6, and 12 months Systemic antibiotics: no Combined peri-implant treatment: no	PPD BoP	The local antibiotic administration reduces peri-implant probing depths and bleeding on probing in patients affected by peri-implantitis, compared to control groups without local antibiotic application
Passarelli, 2021 [42] Antibiotics Systematic review Critically low quality	Studies: n.5 RCT (n.5) Population sample size n.250 Implants n.333 Mean age: MD Gender ratio: MD Dental implants affected Number: MD Position: MD Characteristics: MD Survival: MD	Local antibiotics: yes Type: minocycline, doxycycline Delivery vehicle: microspheres, ointment Systemic antibiotics: yes Type: MTZ Combined peri-implant treatment: no Type: nonsurgical (SRP) and surgical treatment	PPD BoP GI	After 6 months, GI showed a statistically significant improvement in a group treated with local minocycline, compared with the placebo control. After 4 months, PPD and BoP were improved in SRP +minocycline-MTZ group than in SRP alone group. Local antibiotic use can be considered a valid approach to treating peri-implantitis
Wang, 2022 [43] Int J Oral Maxillofac Implants Systematic review and Meta-analysis Low quality	Studies: n.10 RCT (n.7) CS (n.3) Population sample size n.355 Implants n.596 Mean age: 51.5–69.9 y.o. Gender ratio: MD Dental implants affected: MD Position: MD Characteristics: MD Survival: MD	Local antibiotics: yes (3 studies) Type: tetracycline, doxycycline, doxycycline hyclate, minocycline, periocline Regimen: application of local antibiotics subgingivally Systemic antibiotics: yes (7 studies) Type: AZM, AMX, MTZ Regimens: AZM (500 mg/24 h on 1st d and 250 mg/24 h on d 2 to 4) Duration: 4 d; AZM (500 mg/24 h for 3 d before scaling and root planing) Duration: 3 d; AMX (1.5 mg/24 h 3 d preoperatively and 7 d postoperatively) Duration: 10 d; AZM (500 mg/24 h on the d of surgery, and 250 mg/24 h postoperatively for 4 d) Duration: 5 d; MTZ (400 mg/24 h) + AMX (500 mg/8 h) Duration: 14 d Combined peri-implant treatment: yes Type: nonsurgical (SRP) +/− surgical treatment (OFD) Follow-up: 36 months	BoP PPD RBL	The use of adjunctive antibiotics to treat peri-implantitis provided potential benefits in BoP for up to 12 months post-therapy
Oen, M., 2021 [44] BMC Oral Health Systematic review Low quality	Studies: n.9 RCT (n = 2) SR (n = 7) Population sample size MD Implants MD Mean age: MD Gender ratio: MD Dental implants affected Number: MD Position: MD Characteristics: MD Survival: MD	Local antibiotics: no Systemic antibiotics: yes Type: AMX, AZM Combined peri-implant treatment: yes Type: nonsurgical and surgical treatment	BoP PPD PIBL RBL	No strong evidence exists for the use of systemic antibiotics to improve the clinical outcomes in the surgical treatment of peri-implantitis
Toledano-Osorio, 2022 [45] Int. J. Environ. Res. Public Health Systematic review and Meta-analysis Low quality	Studies: n.18 RCT (n.9) PS (n.9) Population sample size n.605 Implants n.870 Mean age: MD Gender ratio: MD Dental implants affected Number: MD Position: MD Characteristics: MD Survival: MD	Local antibiotics: no Systemic antibiotics: yes Type: AZM, AMX, AMX plus MTZ Duration: variable Combined peri-implant treatment: yes Type: nonsurgical (SRP) and surgical treatment Follow-up: 10 d, 1, 6 weeks, 1–3–6–12–36–54 months	PPD BoP PI RBL CAL GI	In the treatment of peri-implantitis, systemic antibiotic somministration did not reduce either PPD nor BoP. A reduction of the clinical attachment level, a lower suppuration and recession, less bone loss, and a reduction in total bacterial counts
Esposito, M., 2004 [46] The Cochrane Database of Systematic Reviews Review Critically low quality	Studies: n.1 RCTs (n.1) Population sample size MD Implants MD Mean age: MD Gender ratio: MD Dental implants affected Number: MD Position: MD Characteristics: MD Survival: MD	Local antibiotics: yes Type: MTZ gel 25% Duration: 12 weeks Systemic antibiotics: yes/no Combined peri-implant treatment: no MTZ vs. nonsurgical (SRP) treatment	PI BoP PPD	No differences were found between the case and the control
Zhao, 2021 [47] Photodiagnosis and Photodynamic therapy Systematic review and Meta-analysis Critically low quality	Studies: n.13 RCT (n.11) N/D (n = 2) Population sample size MD Implants MD Mean age: MD Gender ratio: MD Dental implants affected Number: MD Position: MD Characteristics: MD Survival: MD	Local antibiotics: yes (1/2 local applications) Systemic antibiotics: yes Combined peri-implant treatment: yes Type: nonsurgical (SRP) and surgical treatment	PPD BoP CAL	Meta-analysis outcomes revealed equal clinical evidence for aPDT and antibiotics in periodontitis and peri-implantitis. In addition, aPDT significantly reduced the red complex in both diseases.

Abbreviations: Case series, “CS”; Case-control study, ”CCS”; Randomized control trials, “RCT”; Prospective study, “PS”; Systematic review, “SR”; Male, “M”; Female, “F”; Years old, “y.o.”; Number, “n”; Day(s), “d”; Not defined, “N/D”; Missing data, “MD”; Amoxicillin, “AMX”; Azithromycin, “AZM”; Metronidazole, “MTZ”; Gingival index, “GI”; Plaque index, “PI”; Bleeding on probing, “BoP”; Probing pocket depth, “PPD”; Clinical attachment loss, “CAL”; Radiographic bone loss, “RBL”; Peri-implant bone loss, “PIBL”; Antimicrobial photodynamic therapy, “aPDT”; Photochemotherapy, “PCT”; Scaling and root planing, “SRP”.

**Table 4:** Characteristics of locally-delivered antibiotics administered alone in treating peri-implantitis from included studies.

Authors, Year	Local Antibiotics Regimen	Controls	Outcomes	Conclusion
Toledano, 2021 [41]	Minocycline (“Arestin” in microspheres, “Periocycline” in ointment)	aPDT or Placebo	PPD BoP RBL	PPD and BoP have become reduced after local administration of antibiotics in many cases
	Doxycycline gel (“Atridox”, “Ligosan”) and bone graft “D-Plex 500“	SRP alone (two studies) or No treatment (one study)		
	Lincomycin gel	No treatment		
	Erythromycin gel	No treatment		
	Tetracycline fibers “Actisite”	No treatment		
	MTZ gel “Elyzol”	PCT		
Passarelli, 2021 [42]	Minocycline microspheres	Chlorhexidine 0.1 mL gel 1%	BoP PPD PI	No differences between the groups
Esposito, 2019 [46]	MTZ gel 25% (3 mm subgingivally)	UPD with carbon fiber tip inserted 1–2 mm subgingivally at the lowest power for 15 s	Implant failure	No differences between the two groups

Abbreviations: Photochemotherapy, “PCT”; Antimicrobial photodynamic therapy, “aPDT”; Plaque index, “PI”; Bleeding on probing, “BoP”; Probing pocket depth, “PPD”; “Radiographic bone loss, “RBL”; Scaling and root planing, “SRP”; Ultrasonic periodontal debridement, “UPD”; Metronidazole, “MTZ”.

**Table 5:** Characteristics of locally-delivered antibiotics combined with other interventions in treating peri-implantitis.

Author(s), Year	Local Antibiotics (Regimen) + *Combined Intervention*	Controls	Outcomes	Conclusion
Passarelli, 2021 [42]	Doxycycline hyclate 8.5% + *SRP*	SRP	BoP PPD PI CAL GI	After 4 months, statistically significant differences between groups emerged for the CAL, BoP, and PPD
	Minocycline 10 mg in 0.5 g of ointment + *surgical treatment*	Placebo + SRP		After 6 months, a statistically significant improvement emerged for GI and PPD
	Minocycline ointment ( +/−metronidazole) + *nonsurgical treatment*	SRP		After 4 months, a statistically significant improvement emerged for BoP and PPD
Wang, 2021 [43]	Tetracycline hydrochloride delivery by monolithic ethylene vinyl acetate fiber (for one time of antibiotic with a duration of 10 days) *+ rubber cup polishing + SRP*		PPD BoP	Local antibiotics in peri-implantitis should provide potential benefits in clinical outcomes for up to 12 months after therapy
	Doxycycline “Atridox” subgingivally for one time + *SRP and irrigation with 0.2% CHX*			
	Minocycline “Periocline” applied subgingivally + *OFD at 1, 3, and 6 months*			
Esposito, 2019 [46]	Doxycycline hyclate 8.5% “Atridox” applied through a syringe with a blunt cannula in the peri-implant sulcus + *SRP*	SRP + subgingival irrigation with 0.2% CHX	Implant failure	After 4 months, doxycycline improved CAL and PPD of about 0.6 mm compared to mechanical debridement
Zhao, 2021 [47]	Minocycline gel + *UPD*	SRP/UDP + aPDT	BoP CAL PPD	PPD, BoP, and CAL significantly decreased in the two groups as compared to the baseline but not between the groups
	Minocycline hydrochloride microspheres + *SRP*	SRP + aPDT		
	MTZ 400 mg + AMX 500 mg + *SRP*	SRP + aPDT		

Abbreviations: Photochemotherapy, “PCT“; Antimicrobial photodynamic therapy, “aPDT“; Scaling and root planing, “SRP“; Open flap debridement, “OFD“; Plaque index, “PI“; Bleeding on probing, BoP“; Probing pocket depth, “PPD“; Clinical attachment loss, “CAL“; Gingival index, “GI“; Ultrasonic periodontal debridement, “UPD“; Chlorhexidine, “CHX“; Amoxicillin, “AMX“; Metronidazole, “MTZ”.

**Table 6:** Characteristics of systemically-delivered antibiotics combined with other interventions in the treatment of peri-implantitis.

Author(s), Year	Systemically-delivered Antibiotics (Regimen) + *Combined Intervention*	Controls	Outcomes	Conclusion
Toledano-Osorio, 2022 [45]	AZM (500 mg/24 h at 1st d and 250 mg/24 h for 2–4 d) + *MISD*	MISD + aPDT	BoP PPD	Systemically-delivered antibiotics should be carefully evaluated in peri-implantitis management considering the risk of antibiotic resistance
	AMX (500 mg/8 h) + MTZ (400 mg/24 h for 14 d) + *NSD*	NSD + Placebo		
	AMX (500 mg/8 h for 8 d) + MTZ (400 mg/12 h for 8 d) + porous titanium granule + *OFD*	OFD + antibiotics		
	AMX (500 mg/24 h for 7 d) + MTZ (400 mg/8 h for 7 d) + *OFD*	N/D		
	MTZ (250 mg/8 h for 7 d) + *NSD*	N/D		
	MTZ (500 mg/8 h for 7 d) + *MISD*	N/D		
	AMX (750 mg/12 h) + *MISD*	MISD		
	AZM (250 mg/12 h on the d of surgery + 250 mg/24 h for 4 d) + *OFD*	OFD		
	AMX (500 mg/8 h for 7 d) + MTZ (500 mg/24 h for 7 d) + *MISD*	MISD		
	Clindamycin + MTZ + AZM + tetracicline (for 4 w) + MTZ + AMX + ciprofloxacin + sulfonamide + trimethroprim + MTZ (for 2 w)	N/D		
	AMX 750 mg/12 h for 10 d (3 d prior to surgery) + *OFD + resective techniques*	Resective techniques + antiseptic + OFD		
	AMX (500 mg/8 h for 7 d) + *MISD*	MISD + probiotic		
	AZM (500 mg/24 h for 3 d) + *full mouth SRP*	Full mouth SRP		
	Ornidazole (1.000 mg for 10 d) + *MISD*	N/D		
	AMX (500 mg/8 h for 7 d) + MTZ (400 mg/8 h for 7 d) + +*NSD*	MISD + aPDT		
	Antibiotics N/D (prior to surgery for 1 w, the d of surgery, and 7 d after) + *OFD + bone graft + resorbable membrane*	OFD + bone graft + antibiotic		
	AMX (500 mg/8 h for 7 d) + MTZ (400 mg/8 h for 7 d) + *OFD*	N/D		
	AMX (500 mg/8 h for 5 d) + MTZ (400 mg/8 h for 5 d) + *MISD*	MISD alone		
Wang, 2021 [43]	AZM (500 mg on 1 d and 250 mg on 2 and 4 d) + *SRP + rubber cup polishing*		BoP PPD	Systemic antibiotics, in peri-implantitis, should provide potential benefits in clinical outcomes for up 12 mo. post-therapy
	AZM (500 mg/d for 3 d preoperatively) + *SRP*			
	AMX (1.5 g for 3 d preoperatively and 7 d postoperatively) + *OFD + bone recontouring + rubber cup polishing + CHX 0.2%*			
	MTZ (400 mg/24 h for 14 d) + AMX (500 mg/8 h for 14 d) + *SRP*			
	AZM (500 mg/24 h on the d of surgery + 250 mg/24 h postoperatively for 4 d + *OFD*			
Oen, 2021 [44]	AMX 750 mg/12 h *+ CHX 0.2%*+ *MISD*	No treatment	PPD PIBL	The use of systemic antibiotics as an adjunct to surgical treatment of peri-implantitis did not show beneficial effects
	AZM (250 mg/12 h for 2 d and 250/24 h for 4 d)			
Zhao, 2021 [47]	MTZ (500 mg/24 h) + AMX (500 mg/8 h for 7 d) + *UDP*	UPD + aPDT	BoP CAL PPD	The two groups had a significant decrease in PPD, BoP, and CAL compared to the baseline. Antibiotics reduced PPD, CAL, and BoP after 3 mo. for intergroup comparison
	AMX (375 mg/8 h for 7 d) + MTZ (250 mg/8 h for 7 d) + *SRP*	SRP + aPDT		Antibiotics significantly reduced PPD and CAL for intergroup comparison
	Clarithromycin (500 mg/24 h for 3 d) + *aPDT*	aPDT or aPDT + SRP		Antibiotics significantly reduced PPD for intergroup comparison
	MTZ (400 mg/24 h) + AMX (500 mg/8 h for 7 d) + *SRP*	N/D		A significant decrease in PPD, BoP, and CAL in the two groups was recorded compared to the baseline. aPDT significantly reduced CAL in moderate peri-implant defects in intergroup comparison

Abbreviations: Antimicrobial photodynamic therapy, “aPDT”; Scaling and root planing, “SRP”; Ultrasonic periodontal debridement, “UPD”; Open flap debridement, ”OFD”; Mechanical implant surface debridement, “MISD”; Nonsurgical debridement, “NSD”; Not defined, “N/D”; Chlorhexidine, “CHX”; Amoxicillin, “AMX”; Azithromycin, “AZM”; Metronidazole, “MTZ”; Hour(s), “h”; Day(s), “d”; Week(s), “w”; Month(s), “mo.”; Bleeding on probing, “BoP”; Probing pocket depth, “PPD”; Peri-implant bone loss, “PIBL”; Clinical attachment loss, “CAL”.

**Table 7:** Characteristics of local plus systemic antibiotics administration alone or combined with other interventions in treating peri-implantitis.

Author(s), Year	Local Antibiotics *+/− Combined Intervention*	Systemic Antibiotics *+/− Combined Intervention*	Outcomes	Conclusion
Wang, 2021 [43]	Minocycline “Periocline” + *OFD + SRP at 1, 3, 6 months*	AMX (500 mg/8 h for 3 d)	BoP PPD	Systemic antibiotics in peri-implantitis management should provide benefits in clinical outcomes for up 12 mo. post-therapy
Esposito, 2019 [39]	MTZ 25% gel “Elyzol” + tetracycline hydrochloride “Ambramicine” *+ apically repositioned flap*	AMX (50 mg/kg/d for 8 d) + *SRP before surgery*	PPD CAL REC	There were no baseline imbalances for plaque, marginal bleeding, PPD, CAL, and REC, and no differences after 2 years

Abbreviations: Day(s), “d”; Probing pocket depth, “PPD”; Bleeding on probing, “BoP”; Clinical attachment loss, “CAL”; Recession, “REC”; Amoxicillin, “AMX”; Metronidazole, “MTZ”; Scaling and root planing, “SRP”; Open flap debridement, “OFD”.

## Data Availability

Data supporting reported results can be found in the PROSPERO Registry and the Cochrane Library, BioMed Central, Scopus, and MEDLINE/PubMed databases.
